# Glycopolymers for Antibacterial and Antiviral Applications

**DOI:** 10.3390/molecules28030985

**Published:** 2023-01-18

**Authors:** Ruoyao Mei, Xingyu Heng, Xiaoli Liu, Gaojian Chen

**Affiliations:** 1 Center for Soft Condensed Matter Physics and Interdisciplinary Research, School of Physical Science and Technology, Soochow University, Suzhou 215006, China; 2 Key Laboratory of Polymeric Material Design and Synthesis for Biomedical Function, College of Chemistry, Chemical Engineering and Materials Science, Soochow University, 199 Ren−Ai Road, Suzhou 215123, China

**Keywords:** glycopolymer, antibacterial, antivirus, lectin, inhibition, detection

## Abstract

Diseases induced by bacterial and viral infections are common occurrences in our daily life, and the main prevention and treatment strategies are vaccination and taking antibacterial/antiviral drugs. However, vaccines can only be used for specific viral infections, and the abuse of antibacterial/antiviral drugs will create multi−drug−resistant bacteria and viruses. Therefore, it is necessary to develop more targeted prevention and treatment methods against bacteria and viruses. Proteins on the surface of bacteria and viruses can specifically bind to sugar, so glycopolymers can be used as potential antibacterial and antiviral drugs. In this review, the research of glycopolymers for bacterial/viral detection/inhibition and antibacterial/antiviral applications in recent years are summarized.

## 1. Introduction

In recent decades, a variety of infectious diseases have developed caused by bacteria, including *Staphylococcus aureus*, *Streptococcus haemolyticus* type α, *Streptococcus haemolyticus* type β and viruses including Ebola, Zika, coronavirus (COV) [1]. As a result, different regions are experiencing the spread of diseases caused by these pathogens. With the emergence of superbugs, as well as the global epidemic of COVID−19, we are facing significant challenges in the prevention and treatment of these diseases due to drug resistance and genetic mutations. At present, the effective prevention and treatment strategy for bacterial diseases is to take antibiotic drugs, and the prevention and treatment strategy for viral diseases is mainly vaccination or taking antiviral drugs. However, vaccines can only be used for specific types of viral infections, and antivirals or antimicrobials can make viruses or bacteria resistant. Therefore, it is important to develop more targeted prevention and treatment methods against highly drug−resistant bacteria and viruses. There are a lot of glycoproteins on the surface of bacteria, viruses and other life forms [2]. Different kinds of sugars can specifically combine with pathogens that contain different types of recognition proteins, such as mannose and *Escherichia coli*, L−Fucose and *Vibrio cholerae*, salivary lactose and mumps virus/influenza virus, etc. In addition, the specific binding of sugars and proteins is also affected by a variety of factors, such as the “multivalent effect” of polysaccharides [3,4,5,6], topology [7,8,9] (star−shaped, dendritic), heterogeneity [10,11,12,13,14,15,16], etc. Therefore, glycopolymers with various structures have been developed to specifically recognize and bind with different bacteria and viruses, so as to achieve the application of inhibiting bacterial infection, virus infection, bacterial detection, etc.

In this review, the research on glycopolymers targeting bacteria and viruses in recent years is summarized, and the applications of glycopolymers containing different kinds of sugar side groups, different topological structures and compounds with other materials in bacterial detection, sterilization and antibacterial infection are reviewed in detail. At the same time, the methods of different kinds of glycopolymers to inhibit virus infection by recognizing proteins on the surface of viruses are also summarized and discussed.

## 2. Synthetic Glycopolymers Targeting Bacteria

Fimbriae on the surface of bacteria, such as type 1, type G and type S, contain different types of sugar recognition proteins called lectins, and lectins can specifically bind to different types of sugars, including mannose, galactose, etc. [17]. Therefore, glycopolymers with specific sugar moieties can selectively bind to bacteria. *Escherichia coli* is found to have been studied most comprehensively because of the broad knowledge base available about this organism. At the same time, there are many kinds of lectins on its surface. Therefore, most studies on glycopolymers in the field of bacteria choose *Escherichia coli* as the research object.

### 2.1. Glycopolymers for Bacterial Detection and Capture

**Figure 1:**
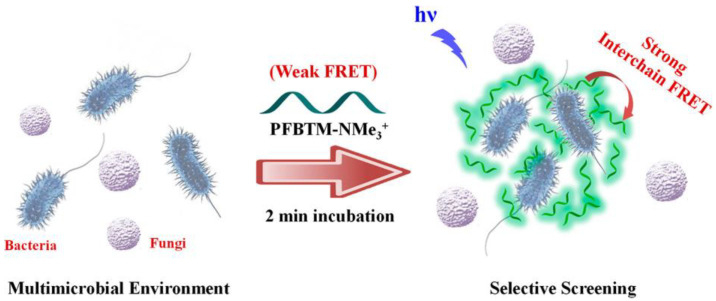
FRET−mediated selective screening and imaging of fungi and bacteria. Figure reproduced from ref. [20] with permission.

Glycopolymers can be used for bacterial capture and detection due to their high affinity to proteins that are on the surface of bacteria. To realize bacterial detection, fluorescence moieties are often introduced into glycopolymers. Disney et al. synthesized mannose−based glycopolymers with fluorescent groups [18]. When mannose recognizes and binds flagellin on the surface of *Escherichia coli* in aqueous solution, a large number of green aggregates will be formed. Aggregation−induced emission (AIE) molecules, such as tetraphenylethylene (TPE), emit fluorescence when their intramolecular rotation is limited by their aggregation state. Li et al. synthesized functional poly(ionic liquid)s (PILs) with both pendent saccharide units and AIE probes [19]. As a result of the electrostatic interactions and sugar−agglutinin reaction, the glycopolymer can be absorbed onto the bacterial surface for the imaging and detection of bacteria. Similarly, Hussain et al. synthesized water−soluble polyfluoroene derivatives containing mannose and quaternary ammonium groups [20]. As shown in Figure 1, the PFBTMNMe_3_^+^ exhibited weak interchain Forster resonance energy transfer (FRET) in water due to the low amount of benzothiadiazole (BT) moiety doping along the backbone. When PFBTM−NMe_3_^+^ binds with the net negative charge on the surface and the mannose binds with lectin FimH, it shows a strong FRET and ratiometric response due to the aggregation of polymers. Through electrostatic interaction and the synergistic effect of the sugar–protein interaction, they can distinguish bacteria and fungi using fluorescence spectroscopy and imaging technology.

**Figure 2:**
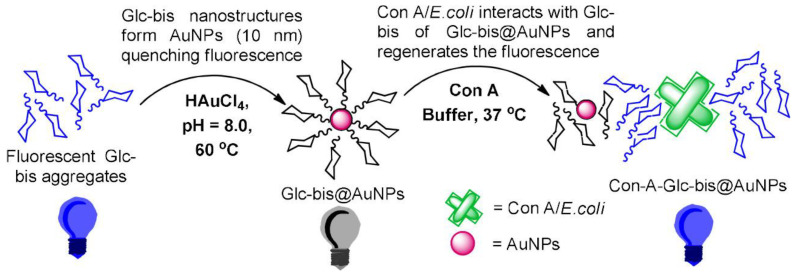
Fluorescence quenching of self−assembled glycopolymers and gold nanoparticles and emission recovery after addition of ConA. Figure reproduced from ref. [22] with permission.

Glycopolymer−conjugated metal or inorganic nanoparticles are also used for bacteria detection. Richards et al. modified the end of poly(ethylene glycol) (PEG) with glucose, and then modified the end−functionalized PEG onto the surface of gold nanoparticles using Au−S bond [21]. Gold nanoparticles modified with glycopolymers can specifically identify *Escherichia coli*. Glycosylated nanoparticles will change their optical properties when they bind with FimH−positive bacteria, so that the absorbance at 700 nm can be monitored with UV−Vis spectroscopy to identify bacterial strains. Similarly, Ajish et al. synthesized glucose−based glycobis(acrylamide) glycopolymer, which can be self−assembled with gold nanoparticles [22]. As shown in Figure 2, the intrinsic fluorescence emission of the self−assembled glycopolymer will be quenched by AuNPs. However, if ConA (Concanavalin A lectin)/*Escherichia coli* is identified, the glycopolymer will be more inclined to combine with ConA/*Escherichia coli,* thus inhibiting the energy transfer between the glycopolymer and gold nanoparticles, and restoring the intrinsic emission of the glycopolymer. The turn−on in the emission intensity can be used for the rapid detection of *Escherichia coli*.

**Figure 3:**
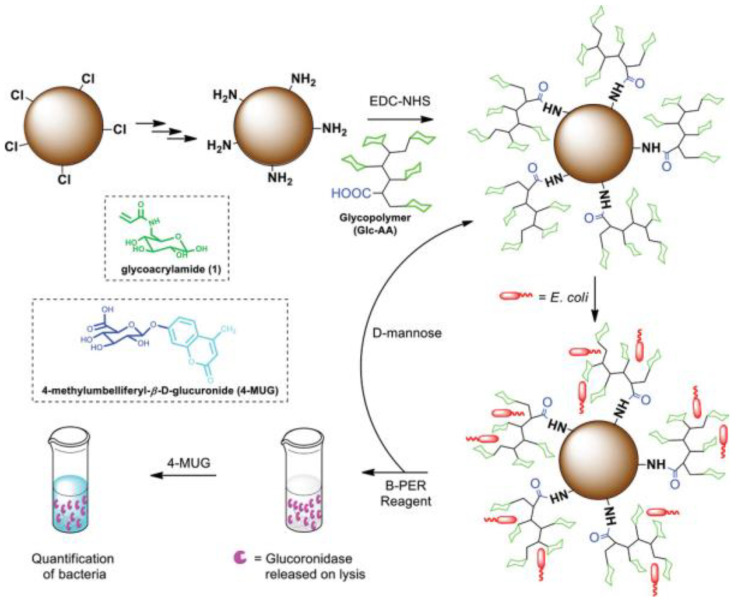
Schematic diagram of the synthesis of a glycopolymer−functionalized resin (Resin−Glc) for capture and quantification of *Escherichia coli*/ConA. Figure reproduced from ref. [26] with permission.

In addition to bacteria detection, glycopolymers conjugated with other materials were fabricated for bacteria capturing. Yang et al. grafted galactose−bearing glycopolymers onto a polypropylene (PP) microfiltration membrane by UV−induced graft copolymerization to obtain a surface that can specifically adhere to *Enterococcus faecalis* [23]. It proved that glycopolymers can be applied to selectively prevent or control bacterial adhesion. Malakootikhah et al. immobilized glucose and/or maltose onto amino MnFe_2_O_4_ @SiO_2_ surface, and those glycopolymers with hyper−crosslinked porous structures can be used for the efficient capture of *bacillus subtilis* (over 99%) [24]. Similarly, Hong et al. prepared magnetic iron oxide nanoparticles immobilized with mannose−based poly(ionic liquid) brushes for the efficient capturing of bacteria, and the nanocomposites also showed expedient recycling capacity and low cytotoxicity [25]. Ajish et al. prepared a glucose−based polymer, which was modified on the resin surface [26] (Figure 3). The “glycocluster effect” presented by the glucose on the resin surface was used to specifically bind *Escherichia coli* or ConA, making the functional resin available for capturing and quantifying *Escherichia coli* or ConA. As mentioned above, conjugating glycopolymers with other materials is a potential strategy for the isolation of pathogens.

### 2.2. Glycopolymers for Sterilization

**Figure 4:**
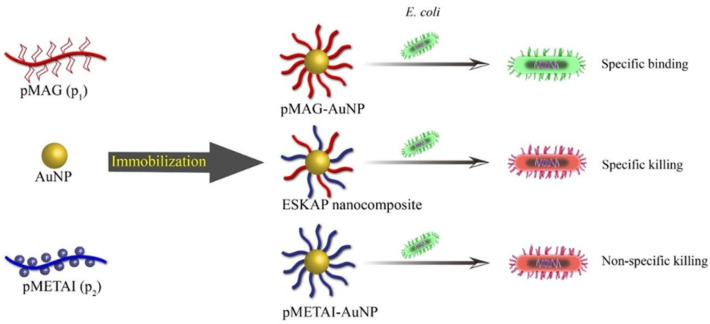
Schematic diagram of the bactericidal and recognition functions of gold nanoparticle complexes regulated by modifying different polymers. Figure reproduced from ref. [29] with permission.

Glycopolymers themselves do not have the ability to kill bacteria, but have the ability to recognize and combine with specific bacteria. Therefore, glycopolymers can be designed and synthesized with antibacterial groups, or conjugated with nanoparticles with bacteria−killing ability to realize the function of sterilization. It is possible for poly(ionic liquid)s (PILs) to approach bacteria due to electrostatic interactions between cationic groups and negatively charged cell walls. Consequently, PILs insert hydrophobic fragments into the membranes of bacteria and cause the rupture and death of bacteria [27,28]. Based on this, Li et al. synthesized mannose−based poly(ionic liquid)s to kill Gram−positive *Staphylococcus aureus* and Gram−negative *Escherichia coli*. [19]. As shown in Figure 4, Yuan et al. modified the surface of gold nanoparticles with poly [2−(methacrylamido)glucopyranose] (pMAG) and poly [2−(methacryloyloxy)ethyl trimethylammonium iodide] (pMETAI) to achieve specific killing of *Escherichia coli* [29]. Quaternary ammonium salt polymers were cationic polymers that destroyed bacterial cell membranes and caused their death after coming into contact with them. The modification not only conferred bactericidal properties to the gold nanoparticles, but also formed a charged protective layer on their surface, maintaining the stability of the nanoparticles under physiological conditions. The glycopolymer not only helped the gold nanoparticles recognize Escherichia coli specifically, but also improved the contact between *Escherichia coli* and the quaternary ammonium salt polymer, thus improving the sterilization efficiency.

**Figure 5:**
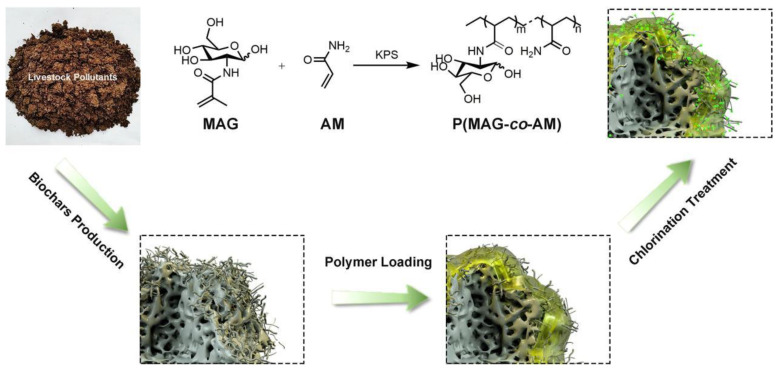
Schematic diagram of the synthesis of BCPMA−Cl for specific killing of *Escherichia coli*. Figure reproduced from ref. [31] with permission.

Biochar is commonly used as a carrier material because of its high surface area, chemical inertness and high stability [30]. Based on this, Borjihan et al. modified biochar with glycopolymer *N*−halamines as an effective *Escherichia coli*−specific−killing agent [31]. As shown in Figure 5, the polymer−loaded biochar was treated with NaClO and acted as an oxidative chlorine (Cl^+^)−bearing agent to inactivate *Escherichia coli*., while the glycopolymer pMAG acted as the specific recognizer of *Escherichia coli* pili protein. Compared with *Staphylococcus* aureus, the modified biochar has a higher specific killing capacity against *Escherichia coli*.

**Figure 6:**
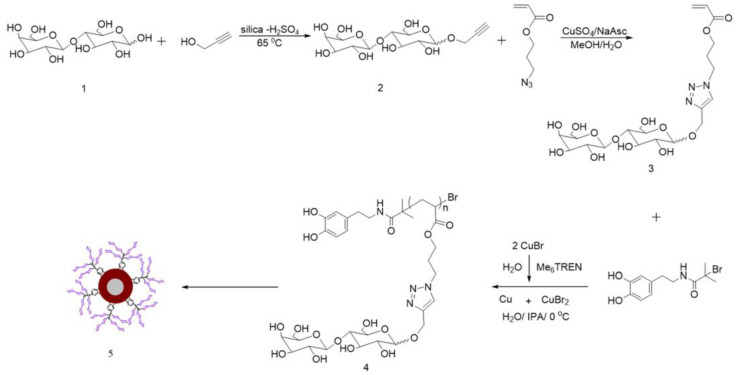
Synthetic route of Fe_3_O_4_@TiO_2_@poly(LacA) hybrid nanoparticles. Figure reproduced from ref. [33] with permission.

After being exposed to sunlight, photocatalysts, such as titanium dioxide (TiO2), can produce reactive oxygen species to drive the disinfection process [32], and further destroy the cell membrane/cell wall of bacteria, thus inactivating pathogens. Wang et al. synthesized a lactose−based glycopolymer through copper (0)−mediated living radical polymerization (Cu(0)−LRP) [33] (Figure 6). After being modified onto the surface of magnetic nanoparticles, the glycopolymer can be used to capture *Escherichia coli*, and *Escherichia coli* can be killed by TiO_2_. With the help of lactose−based glycopolymer, Wang et al. prepared magnetic nanoparticle composites that can capture and specifically kill *Escherichia coli* from aqueous solution.

**Figure 7:**
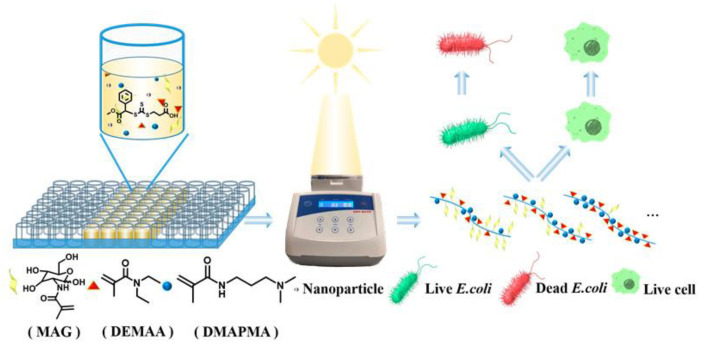
High−throughput open polymerization to prepare glycopolymers containing hydrophobic fragments and cations [47].

In addition to the above metal and inorganic materials, some organics also have the bactericidal effect, and glycopolymers can also be compounded with those materials. Antimicrobial peptides (AMPs) are widely used in antibacterial applications and are effective at killing bacteria [34,35,36,37,38,39,40,41,42,43,44,45]. Pranantyo et al. used atom transfer radical polymerization, N−carboxyanhydride ring−opening polymerization, and copper−catalyzed azide–alkyne “click” cycloaddition techniques to combine mannose, glucose and galactose glycopolymers with antibacterial peptides to form a four−armed star structure [46]. While two α−polylysine arms were capable of killing bacteria, the other two glycopolymer arms could moderate their toxicity to mammalian cells and improve their binding affinity to bacteria. Proper ratios of cationic and hydrophobic moieties in AMPs are key for their function. By mimicking AMP, synthetic glycopolymers show promising application potential with proper molecular design. Zheng et al. prepared antibacterial glycopolymers containing cationic and hydrophobic fragments through high throughput technique and recyclable−catalyst−aided, opened−to−air, and sunlight−photolyzed reversible addition–fragmentation chain transfer (ROS−RAFT) polymerization to simulate the antibacterial principle of antibacterial peptides [47]. As shown in Figure 7, a combination of cationic, hydrophobic fragments and sugar can be optimized through high−throughput screening to achieve bactericidal performance with low cytotoxicity. Among them, polymers with high N−[3−(dimethylamino)propyl] methacrylamide (DMAPMA) content had significant antibacterial ability against *Escherichia coli* and *Staphylococcus aureus*, while polymers with more MAG had higher killing ability against *Escherichia coli* compared with *Staphylococcus aureus* due to their specific identification with *Escherichia coli*. By tuning polymer composition, the modification of glycopolymers allows materials to identify specific bacteria and kill them more efficiently.

### 2.3. Glycopolymers for Inhibiting Bacterial Infection

The specific high affinity between lectins on bacterial pili and polysaccharides on the surface of host cells determines the adhesion and continuous proliferation of bacteria in specific host cells. For example, *Escherichia coli* with type 1 fimbriae contains a lectin (FimH protein) that can specifically bind to mannose, and type 1 fimbria can enhance the persistence of *Escherichia coli* bacteria and the inflammatory response after infection, thereby enhancing urinary tract infection [48]; *Escherichia coli* with type P pili express lectin specifically binding to galactose at the end of pili. Through the interaction of lectin and galactose groups on cell surface, *Escherichia coli* will multiply on the surface of kidney cells, causing pyelonephritis [49]. Glycopolymers with higher affinity to lectins can preferentially bind to bacteria and occupy the binding sites on the bacterial surface, making it impossible for bacteria to adhere to the cell surface, so as to achieve the purpose of inhibiting bacterial infection. Therefore, we can inhibit bacterial infections without killing them.

**Figure 8:**
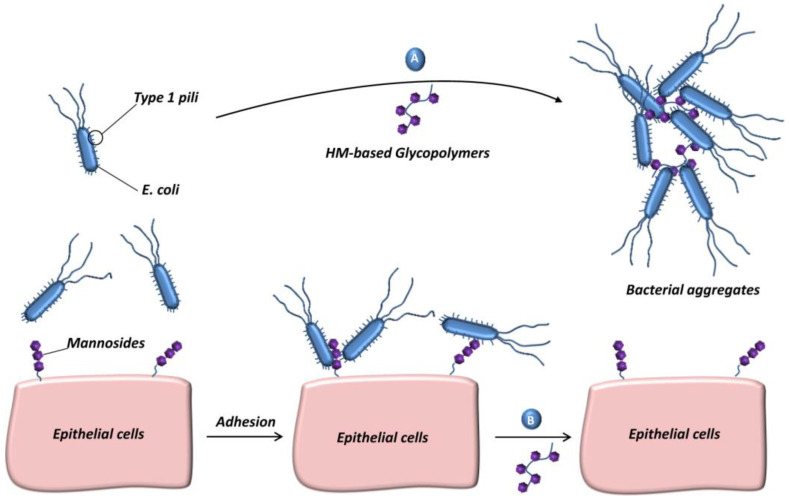
HM−based glycopolymers for inhibiting bacterial infection are shown in figure. (**A**) sequestration of free bacteria in the lumen of the gut and (**B**) disruption of established *Escherichia coli*−cell interactions. Figure reproduced from ref. [50] with permission.

Yan et al. synthesized an n−heptyl α−D−mannose(HM)−based glycopolymer [50]. Due to the specific binding effect of mannose and *Escherichia coli*, the glycopolymer can not only occupy the mannose−specific adhesion site of free bacteria (Figure 8A), but also compete with cells that have already combined with *Escherichia coli*, thus breaking the established interaction between bacteria and intestinal epithelial cells (Figure 8B) and inhibiting the adhesion and proliferation of *Escherichia coli* on cells.

In addition to the types of sugar units, there are many other factors that can enhance the ability of glycopolymers to combine with bacteria. Xue et al. synthesized heteroglycopolymers (PM−GM) and homoglycopolymers (PM−GG and PM−MM) via Ugi reaction and click chemistry [16]. Subsequently, the binding constant Ka with Con A was calculated by Langmuir model. The Ka of PM−GM was 2.09, while the Ka of PM−GG and PM−MM were 1.36 and 1.62. It proved that heteroglycopolymer PM−GM has the best binding ability, owing to the heterocluster effect generated by heteromultivant ligands. Meanwhile, bacteria adhesion studies also demonstrated that the heterocluster effect made a stronger binding with fimH.

**Figure 9:**
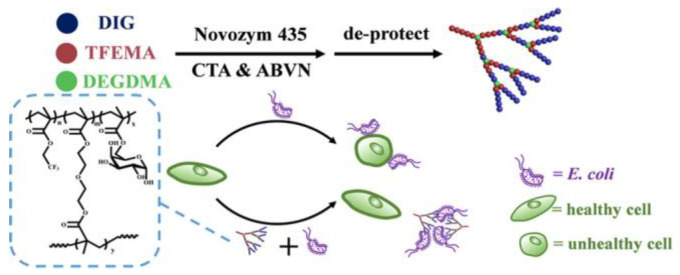
Binding of hyperbranched glycopolymer to *Escherichia coli* [51].

The structure of glycopolymers also has a significant impact on the ability to bind with bacteria. Zheng et al. combined enzymatic monomer transformation with reversible addition–fragmentation chain transfer (RAFT) polymerization to synthesize gradient glycopolymers with hyperbranched structure through a one−pot method [51]. At the same time, gradient linear analogues and block glycopolymers were synthesized for comparison. The results showed that, compared with linear analogues and block glycopolymers, hyperbranched glycopolymers had the most significant bacterial binding ability (Figure 9). As a result, the hyperbranched glycopolymer has a significant inhibitory effect on bacterial infection.

**Figure 10:**
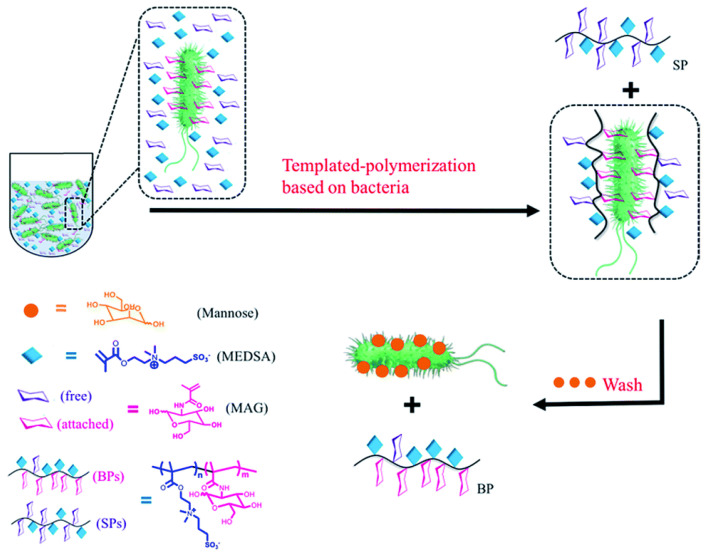
Two different polymers are obtained by polymerization using bacteria as living templates: (1) in solution (SP), (2) on the bacterial surface (BP) [52].

Usually, synthetic glycopolymers can bind with more than one kind of proteins, but we need other strategies to enhance specificity and obtain glycopolymers that can recognize bacteria more specifically. Luo et al. proposed a method to prepare highly specific glycopolymers using bacteria as the “living” template [52] (Figure 10). Through this bacteria−sugar monomer−adaptation−polymerization (BS−MAP) method, glycopolymers obtained from the bacterial surface can recognize *Escherichia coli* at the strain level. The specific bacterial binding ability of the glycopolymer was confirmed by bacterial aggregation experiment and quartz crystal microbalance with dissipation. Furthermore, the anti−infection experiment and co−culture experiment showed that the synthesized glycopolymer effectively inhibits bacterial infection.

## 3. Glycopolymers Targeting Viruses

There are a large number of sugars on the surface of cells, which facilitate the transmission of biological information between cells by interacting with specific proteins. In contrast, pathogens also recognize their specific host cells through glycoprotein interactions, that is, glycorecognition proteins (lectins) on the surface of pathogens interact with sugar units on the cell surface to cause viral infection. Based on these interaction mechanisms, the antiviral application of sugar has been heavily studied to develop specific targeting systems that can act as inhibitors of the virus. This section summarized two common strategies that have been studied in recent years to prevent virus infection: targeting cell surface proteins and targeting virus surface proteins.

### 3.1. Glycopolymers Targeting Cell Surface Proteins

C−type lectin receptor, dendritic cell−specific intercellular adhesion molecule 3 grabbing nonintegrin (DC−SIGN), is a pattern recognition receptor expressed on macrophages and dendritic cells. It has been identified as a receptor for many pathogens, such as SARS−CoV−2 and HIV. These viruses spread and escape through the binding of DC−SIGN captured by sugar molecules on their surface and dendritic cell−specific ICAM−3 [53]. In the context of the recent SARSCoV−2 epidemic, DC−SIGN−mediated viral transmission and innate immune responses have been identified as a potential factor in the pathogenesis of COVID−19 [54]. Therefore, the design of glycopolymers with stronger binding capacity to DC−SIGN to inhibit viral binding to DC−SIGN is an attractive strategy to attenuate excessive innate immune responses and prevent disease progression.

**Figure 11:**
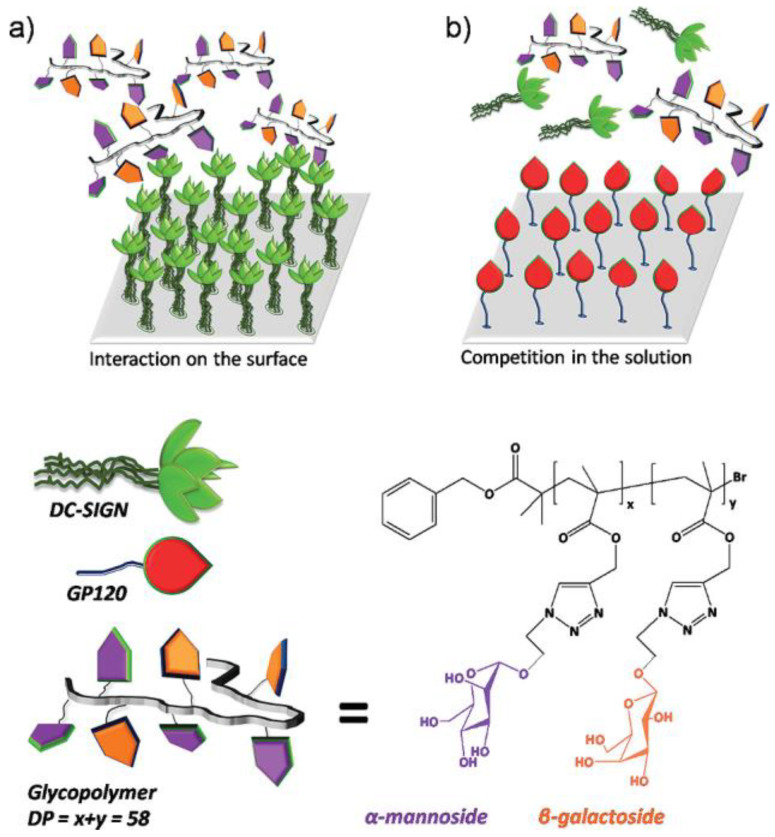
(**a**) DC−SIGN−functionalized surfaces were used to evaluate the binding affinity of glycocopolymers; (**b**) gp120−functionalized surfaces are used for competitive binding studies. (Bottom) Schematic diagram of DC−SIGN and gp120 structure and chemical structure of glycocopolymer. Figure reproduced from ref. [55] with permission.

The ability of glycopolymers binding to DC−SIGN is influenced by multiple factors. Different types and structures of sugars have different degrees of binding capacity to the virus. In addition, we can also strengthen the binding ability to virus by simulating the glycoproteins. Becer et al. synthesized a mannose−based glycopolymer using copper−mediated living radical polymerization and azide−alkyne [3 + 2] Huisgen cycloaddition reaction to interact with DC−SIGN and inhibit the binding of HIV envelope glycoprotein gp120 to DC−SIGN [55]. The binding affinity of the glycocopolymer to DC−SIGN was investigated by multi−channel surface plasmon resonance (MC−SPR). They used a DC−SIGN functionalized surface to evaluate the binding affinity of glycopolymers (Figure 11a), and used gp120 functionalized surfaces for competitive binding studies (Figure 11b). It proved that the increase in mannose content was the key to high affinity; by contrast, the increase in galactose density decreased the affinity for DC−SIGN.

**Figure 12:**
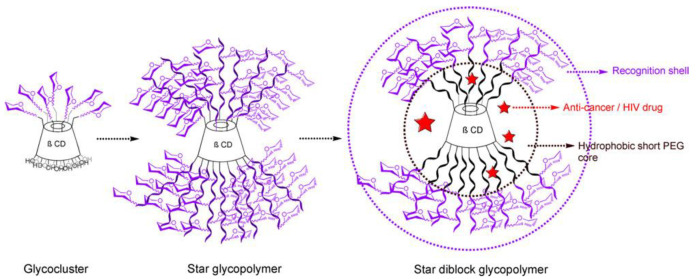
Sugar clusters, star type glycopolymers and star block type glycopolymers. Figure reproduced from ref. [56] with permission.

Zhang et al. synthesized a series of mannose−based cyclodextrin glycopolymers, including sugar clusters and star sugar copolymers, by CuAAC Huisgen coupling and copper−mediated living radical polymerization [56]. These glycoconjugates exhibit high binding affinity to the lectin DC−SIGN, so that these glycopolymers act as an inhibitor to prevent the binding of the HIV envelope protein gp120 to DC−SIGN at nanomolar concentrations. In addition, they also prepared star block glycopolymers and constructed an intelligent drug delivery system with sugar recognition sites, showing application prospects in HIV treatment and intelligent drug delivery (Figure 12).

**Figure 13:**
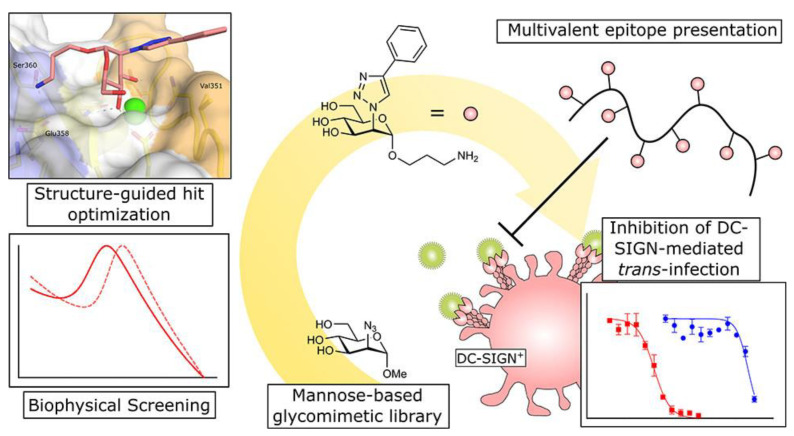
Interaction of triazole−based mannose analogues with DC−SIGN cells. Figure reproduced from ref. [53] with permission.

Recently, Cramer et al. designed a mannose−modified poly−L−lysine complex [53]. Due to the polyvalent effect of sugar, the complex inhibited the binding of SARS−CoV−2 spike protein to DC−SIGN on the cell surface (Figure 13). As a result, the binding amount of cells pre−incubated with the complex to the virus expressing SARS−CoV−2 spike protein is greatly reduced, which limits the spread of the virus between different cells.

### 3.2. Glycopolymers Targeting Virus Surface Lectin

**Figure 14:**
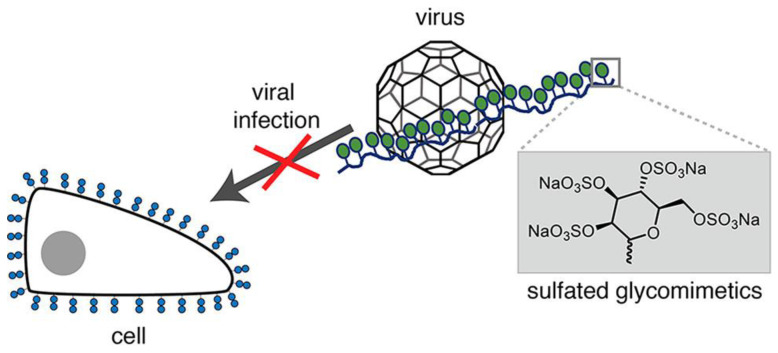
A model of competition between sulfate−like polymers and cellular glycans to inhibit virus invasion. Figure reproduced from ref. [66] with permission.

It is difficult to produce vaccines that are effective against multiple existing and emerging strains of viruses due to virus mutations [57,58,59]. To prevent infection, preventing the virus from attaching to the surface of the cell is the general approach to preventing infection with many viruses, including influenza [60,61,62]. Viruses can adhere to cells by binding to glycans on the cell surface; however, by regulating sugar species, density, topology, etc., glycopolymers can have a stronger virus−binding capacity. Therefore, using glycopolymers to recognize viruses can inhibit viral binding to cells. Viruses can recognize various glycans, such as glycans terminating sialic acids (Neu5Ac), and glycosaminoglycans (GAGs), such as heparan sulfates (HS). However, the use of natural polysaccharides as inhibitors for virus recognition may present many challenges to the safety of clinical applications [63,64]. Firstly, the molecules are heterogeneous. The preparations may be mixed with glycans and contain a variety of impurities. Secondly, as natural glycans, they may also cause biological side effects due to their inconsistent quality and traces of contamination. In search of substitutes, synthetic glycomimetics provide the compound more controllability over its structure. Glycomimetics have been shown to improve stability, bioavailability and half−life. Moreover, the activities of glycomimetics are comparable or even higher than their corresponding natural polysaccharides [65]. Based on this, Soria−Martinez et al. synthesized highly sulfated synthetic glycomimetics designed to mimic heparin and other natural polysaccharides with high sulfation degree as viral binding/infection inhibitors (Figure 14). The synthetic glycomimetics can effectively inhibit human papillomavirus (HPV16) infection in vitro and maintain the antiviral activity in vivo [66].

**Figure 15:**
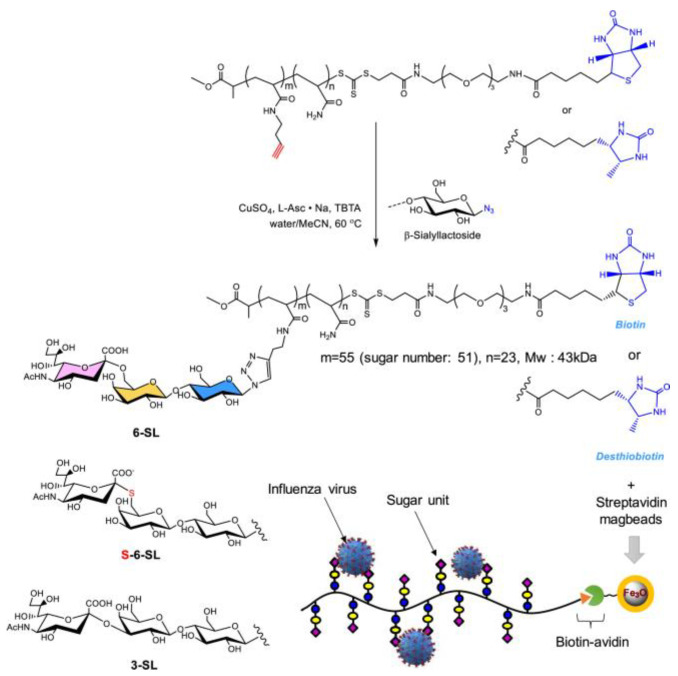
Chemical structures and schematic procedure for the preparation of the glyco−magbeads. Figure reproduced from ref. [69] with permission.

In the molecular basis of influenza virus attaching to cell surface, the viral membrane protein hemagglutinin (HA) binds to the terminal sialic acid residues of cell surface glycoprotein. Therefore, inhibition of this interaction can effectively prevent influenza virus infection. Based on this, Watson et al. synthesized a glycopolymer of sialic acid and acrylamide, which combined sialic acid with HA to produce a blocking effect on the virus [67]. In addition, the glycopolymer can also block the contact between viruses and cells by steric effect. Similarly, Liu et al. prepared glycopolymers by reacting monomeric S−sialoside with polymers that contained maleic anhydride moieties [68]. Hemagglutinin and neuraminidase interact multivalently with mucin mimic glycoconjugates to inhibit viral infection. In addition, Li et al. synthesized sialyllactose−based glycopolymers by RAFT polymerization using a biotinylated chain transfer agent (CTA) and “post−click” chemistry [69] (Figure 15). The glyco−magbeads were obtained by reacting the biotinylated glycopolymers with Streptavidin magbeads, and they can be used to selectively capture influenza viruses.

**Figure 16:**
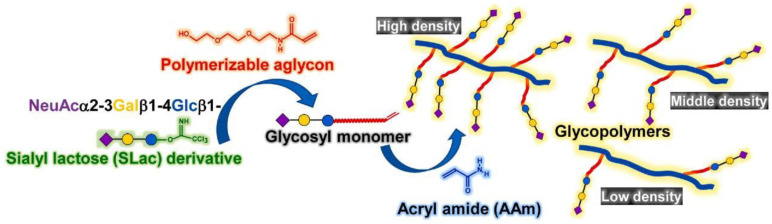
Construction of a synthetic scheme for multivalent glycopolymer containing SLac part. Figure reproduced from ref. [71] with permission.

The blocking effect is influenced by a variety of parameters. In addition to the binding of specific sugars to viruses, the sugar density also affects their binding ability to viruses. Nagao et al. synthesized glycopolymers bearing sialyllactoses by “post−click” chemistry and evaluated their interaction with the influenza virus, and they proved that glycohomopolymers are not always the best structures, and the sugar density must be appropriate to exhibit a cluster glycoside effect as a result of a potential lack of flexibility [70]. Moreover, hemagglutinin has three binding sites on the surface, and the interaction between glycopolymers and the influenza virus will be enhanced if the polymer length is longer than the distance between the binding sites of hemagglutinin. Matsuoka et al. prepared sialyl α2 → 3 lactose(Slac)−based glycopolymer with acryl amide (AAm) as regulator for the arrangement of sugar density, and the glycopolymers showed inhibitory effects on the mumps virus [71] (Figure 16). The results showed that the glycopolymer with the lowest sugar density had the highest inhibitory capacity among three kinds of glycopolymers with 100%, 71.5% and 41% sugar contents. The results suggest that an appropriate distance between a sugar residue and the adjacent sugar residue in the glycopolymer is needed as the glycopolymer with the 41% sugar content is the most effective in binding MuV−HNs on the viral surface.

**Figure 17:**
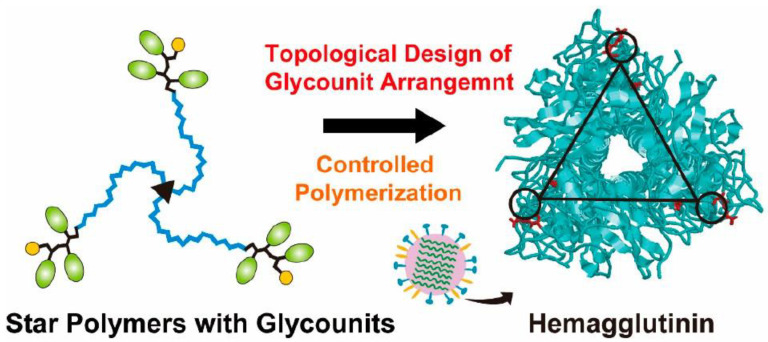
Topological design of star polymers with glycounits (**left**) and hemagglutinin (**right**). Figure reproduced from ref. [72] with permission.

In addition, the topology of the glycopolymers has a significant impact on the binding ability of viruses. As shown in Figure 17, Nagao et al. synthesized a three−arm star−shaped glycopolymer containing sialyllactose based on the structure of HA with three sugar−binding pockets [72]. Precisely synthesized glycopolymers recognize HA on the surface of influenza viruses and interact with influenza H3N2. Among them, the interaction of the star copolymers with HA depends on the length of the polymer arm. When the hydrodynamic diameter of star copolymer and the distance between sugar−binding pockets of HA were comparable, the interaction of the star glycopolymers with HA was maximized.

## 4. Conclusions and Outlook

Glycopolymers have been widely studied in the fields of bacterial detection and capture, inhibiting bacterial infection, inhibiting viral infection and so on. Additionally, glycopolymers may be applied to detect the type of pathogen or to perform precise treatment without affecting the intestinal flora for practical applications. Glycopolymers have the ability to recognize proteins specifically, and the binding of glycopolymers with bacteria and viruses can be improved and regulated by varying sugar type, sugar density and topological structure. A variety of sugars, such as mannose and glucose, have been chosen to synthesize glycopolymers that identify bacteria. The current reported articles show great potential for the application of glycopolymers in the treatment of bacterial and viral infection, although most reports are still very preliminary. Moreover, the use of glycopolymers is unnecessary if broad−spectrum sterilization is needed. It should be noted that many bacteria have similar sugar−binding sites towards certain sugars. For example, both *Escherichia coli* and *Salmonella* spp. have binding sites toward mannose. In order to increase specificity, polymer chain composition, sequence and structure need to be tailored for different bacteria. The cytotoxicity of glycopolymers is another parameter that needs to be considered. Mammalian membranes are rich in cholesterol and composed primarily of lipids with a lower net charge compared to bacterial membranes [73]. Therefore, glycopolymers with high antibacterial activity and low cytotoxicity are required based on the different natures of bacterial and mammalian cell membranes. It is worth noting that most synthetic antibacterial glycopolymers reported in the literature are nondegradable and might stimulate immune cells to elicit some unknown immune effects, which limits their application in vivo. Inactive and low−toxic polymers may reduce the possibility of bacteria becoming resistant to them as well as the accumulation of cytotoxicity in their environment. In addition, new elements, such as selenium [74] and molecular “needles” or “razors” [75], can be incorporated into the glycopolymer chain for enhanced antibacterial performance. Regarding the application in the field of inhibiting virus infection, the reported strategies are divided into two main types: 1. Binding to virus directly. For example, sialyllactose−containing polymers can bind to influenza virus and mumps virus. 2. Binding to host cells that are the targets of the virus, so the virus cannot compete with the glycopolymer to bind to the host cells. For example, mannose−based glycopolymers can bind to DC−SIGN on the surface of dendritic cells, which is usually a target of HIV. Glycopolymers with enhanced specificity and appropriate polymer chain composition, sequence and structure are required. Another important future direction may be host−directed immunotherapy, as the disadvantages of pathogen−directed therapeutics include resistance selection and virus mutation. The effectiveness of cancer immunotherapy has aroused people’s great interest in applying these methods to treat bacterial and viral infections [74,75,76,77,78]. Glycopolymers have shown potential in cancer immunotherapy [79,80], which is believed to be a good candidate for bacterial and viral immunotherapy treatment with proper molecular design.

## Data Availability

Not applicable.

## References

[1] Dhama K., Khan S., Tiwari R., Sircar S., Bhat S., Malik Y.S., Singh K.P., Chaicumpa W., Bonilla-Aldana D.K., Rodriguez-Morales A.J. (2020). Coronavirus disease 2019-COVID-19. Clin. Microbiol. Rev..

[2] Miura Y., Hoshino Y., Seto H. (2016). Glycopolymer Nanobiotechnology. Chem. Rev..

[3] Behren S., Westerlind U. (2022). Novel approaches to design glycan-based antibacterial inhibitors. Eur. J. Org. Chem..

[4] Mammen M., Choi S.K., Whitesides G.M. (1998). Polyvalent interactions in biological systems: Implications for design and use of multivalent ligands and inhibitors. Angew. Chem. Int. Ed..

[5] Kiessling L.L., Pontrello J.K., Schuster M.C., Wong C.-H. (2003). Synthetic multivalent carbohydrate ligands as effectors or inhibitors of biological processes. Carbohydrate-Based Drug Discovery.

[6] Kiessling L.L., Gestwicki J.E., Strong L.E. (2000). Synthetic multivalent ligands in the exploration of cell-surface interactions. Curr. Opin. Chem. Biol..

[7] Ting S.R.S., Chen G.J., Stenzel M.H. (2010). Synthesis of glycopolymers and their multivalent recognitions with lectins. Polym. Chem..

[8] Gestwicki J.E., Cairo C.W., Strong L.E., Oetjen K.A., Kiessling L.L. (2002). Influencing receptor-ligand binding mechanisms with multivalent ligand architecture. J. Am. Chem. Soc..

[9] Becer C.R. (2012). The Glycopolymer Code: Synthesis of glycopolymers and multivalent carbohydrate-Lectin interactions. Macromol. Rapid Commun..

[10] Ladmiral V., Mantovani G., Clarkson G.J., Cauet S., Irwin J.L., Haddleton D.M. (2006). Synthesis of neoglycopolymers by a combination of “click chemistry” and living radical polymerization. J. Am. Chem. Soc..

[11] Nishida Y., Uzawa H., Toba T., Sasaki K., Kondo H., Kobayashi K. (2000). A facile synthetic approach to L- and P-selectin blockers via copolymerization of vinyl monomers constructing the key carbohydrate modules of sialyl lewisX mimics. Biomacromolecules.

[12] Gomez-Garcia M., Benito J.M., Butera A.P., Mellet C.O., Fernandez J.M.G., Blanco J.L.J. (2012). Probing carbohydrate-lectin recognition in heterogeneous environments with monodisperse cyclodextrin-based glycoclusters. J. Org. Chem..

[13] Gomez-Garcia M., Benito J.M., Rodriguez-Lucena D., Yu J.X., Chmurski K., Mellet C.O., Gallego R.G., Maestre A., Defaye J., Fernandez J.M.G. (2005). Probing secondary carbohydrate-protein interactions with highly dense cyclodextrin-centered heteroglycoclusters: The heterocluster effect. J. Am. Chem. Soc..

[14] Vico R.V., Voskuhl J., Ravoo B.J. (2011). Multivalent interaction of cyclodextrin vesicles, carbohydrate guests, and lectins: A kinetic investigation. Langmuir.

[15] Liang C.H., Wang S.K., Lin C.W., Wang C.C., Wong C.H., Wu C.Y. (2011). Effects of neighboring glycans on antibody-carbohydrate interaction. Angew. Chem. Int. Ed..

[16] Xue L.L., Xiong X.H., Chen K., Luan Y.F., Chen G.J., Chen H. (2016). Modular synthesis of glycopolymers with well-defined sugar units in the side chain via Ugi reaction and click chemistry: Hetero vs. homo. Polym. Chem..

[17] Sharon N. (1987). Bacterial lectins, cell-cell recognition and infectious disease. FEBS Lett..

[18] Disney M.D., Zheng J., Swager T.M., Seeberger P.H. (2004). Detection of bacteria with carbohydrate-functionalized fluorescent polymers. J. Am. Chem. Soc..

[19] Li D., Chen J., Hong M., Wang Y., Haddleton D.M., Li G.Z., Zhang Q. (2021). Cationic glycopolymers with aggregation-induced emission for the killing, imaging, and detection of bacteria. Biomacromolecules.

[20] Hussain S., Lv F., Qi R., Senthilkumar T., Zhao H., Chen Y., Liu L., Wang S. (2020). Forster resonance energy transfer mediated rapid andsynergistic discrimination of bacteria over fungi using a cationic conjugated glycopolymer. ACS Appl. Bio Mater..

[21] Richards S.J., Fullam E., Besra G.S., Gibson M.I. (2014). Discrimination between bacterial phenotypes using glyco-nanoparticles and the impact of polymer coating on detection readouts. J. Mater. Chem. B.

[22] Ajish J.K., Kanagare A.B., Kumar K.S.A., Subramanian M., Ballal A.D., Kumar M. (2019). Self-assembled glycobis(acrylamide)-stabilized gold nanoparticles for fluorescent turn-on sensing of lectin and *Escherichia coli*. ACS Appl. Nano Mater..

[23] Yang Q., Strathmann M., Rumpf A., Schaule G., Ulbricht M. (2010). Grafted glycopolymer-based receptor mimics on polymer support for selective adhesion of bacteria. ACS Appl. Mater. Interfaces.

[24] Malakootikhah J., Rezayan A.H., Negahdari B., Nasseri S., Rastegar H. (2018). Porous MnFe2O4@SiO2 magnetic glycopolymer: A multivalent nanostructure for efficient removal of bacteria from aqueous solution. Ecotoxicol. Environ. Saf..

[25] Hong M., Miao Z., Xu X., Zhang Q. (2020). Magnetic iron oxide nanoparticles immobilized with sugar-containing poly(ionic liquid) brushes for efficient trapping and killing of bacteria. ACS Appl. Bio Mater..

[26] Ajish J.K., Abraham H.M., Subramanian M., Kumar K.S.A. (2021). A reusable column method using glycopolymer-functionalized resins for capture-detection of proteins and *Escherichia coli*. Macromol. Biosci..

[27] Li X., Bai H.T., Yang Y.C., Yoon J., Wang S., Zhang X. (2019). Supramolecular antibacterial materials for combatting antibiotic resistance. Adv. Mater..

[28] Chen J., Wang F.Y.K., Liu Q.M., Du J.Z. (2014). Antibacterial polymeric nanostructures for biomedical applications. Chem. Commun..

[29] Yuan Y.Q., Liu F., Xue L.L., Wang H.W., Pan J.J., Cui Y.C., Chen H., Yuan L. (2016). Recyclable *Escherichia coli*-specific-killing AuNP-polymer (ESKAP) nanocomposites. ACS Appl. Mater. Interfaces.

[30] Li L., Zhang K., Chen L., Huang Z., Liu G., Li M., Wen Y. (2017). Mass preparation of micro/nano-powders of biochar with water-dispersibility and their potential application. New J. Chem..

[31] Borjihan Q., Yao Q.F., Qu H.H., Wu H.X., Liu Y., Dong A. (2021). Glycopolymer N-halamine-modified biochars with high specificity for *Escherichia coli* eradication. Chin. J. Chem. Eng..

[32] Dalrymple O.K., Stefanakos E., Trotz M.A., Goswami D.Y. (2010). A review of the mechanisms and modeling of photocatalytic disinfection. Appl. Catal. B-Environ..

[33] Wang B., Shang C., Miao Z., Guo S., Zhang Q. (2021). Lactose-containing glycopolymer grafted onto magnetic titanium dioxide nanomaterials for targeted capture and photocatalytic killing of pathogenic bacteria. Eur. Polym. J..

[34] Tew G.N., Liu D.H., Chen B., Doerksen R.J., Kaplan J., Carroll P.J., Klein M.L., DeGrado W.F. (2002). De novo design of biomimetic antimicrobial polymers. Proc. Natl. Acad. Sci. USA.

[35] Crespo L., Sanclimens G., Pons M., Giralt E., Royo M., Albericio F. (2005). Peptide and amide bond-containing dendrimers. Chem. Rev..

[36] Mowery B.P., Lee S.E., Kissounko D.A., Epand R.F., Epand R.M., Weisblum B., Stahl S.S., Gellman S.H. (2007). Mimicry of antimicrobial host-defense peptides by random copolymers. J. Am. Chem. Soc..

[37] Gabriel G.J., Maegerlein J.A., Nelson C.E., Dabkowski J.M., Eren T., Nusslein K., Tew G.N. (2009). Comparison of facially amphiphilic versus segregated monomers in the design of antibacterial copolymers. Chem. Eur. J..

[38] Giuliani A., Rinaldi A.C. (2011). Beyond natural antimicrobial peptides: Multimeric peptides and other peptidomimetic approaches. Cell. Mol. Life Sci..

[39] Kuroda K., Caputo G.A. (2013). Antimicrobial polymers as synthetic mimics of host-defense peptides. WIREs Nanomed. Nanobiotechnol..

[40] Muñoz-Bonilla A., Cerrada M.L., Fernández-García M., Muñoz-Bonilla A., Cerrada M., Fernández-García M. (2014). Introduction to Antimicrobial Polymeric Materials. Polymeric Materials with Antimicrobial Activity: From Synthesis to Applications.

[41] Stach M., Siriwardena T.N., Kohler T., van Delden C., Darbre T., Reymond J.L. (2014). Combining topology and sequence design for the discovery of potent antimicrobial peptide dendrimers against multidrug-resistant pseudomonas aeruginosa. Angew. Chem. Int. Ed..

[42] Liu R.H., Chen X.Y., Chakraborty S., Lemke J.J., Hayouka Z., Chow C., Welch R.A., Weisblum B., Masters K.S., Gellman S.H. (2014). Tuning the biological activity profile of antibacterial polymers via subunit substitution pattern. J. Am. Chem. Soc..

[43] Lam S.J., O’Brien-Simpson N.M., Pantarat N., Sulistio A., Wong E.H.H., Chen Y.-Y., Lenzo J.C., Holden J.A., Blencowe A., Reynolds E.C. (2016). Combating multidrug-resistant Gram-negative bacteria with structurally nanoengineered antimicrobial peptide polymers. Nat. Microbiol..

[44] Namivandi-Zangeneh R., Kwan R.J., Nguyen T.-K., Yeow J., Byrne F.L., Oehlers S.H., Wong E.H.H., Boyer C. (2018). The effects of polymer topology and chain length on the antimicrobial activity and hemocompatibility of amphiphilic ternary copolymers. Polym. Chem..

[45] Judzewitsch P.R., Nguyen T.-K., Shanmugam S., Wong E.H.H., Boyer C. (2018). Towards sequence-controlled antimicrobial polymers: Effect of polymer block order on antimicrobial activity. Angew. Chem. Int. Ed..

[46] Pranantyo D., Xu L.Q., Hou Z., Kang E.-T., Chan-Park M.B. (2017). Increasing bacterial affinity and cytocompatibility with four-arm star glycopolymers and antimicrobial α-polylysine. Polym. Chem..

[47] Zheng Y., Luo Y., Feng K., Zhang W., Chen G. (2019). High throughput screening of glycopolymers: Balance between cytotoxicity and antibacterial property. ACS Macro Lett..

[48] Connell I., Agace W., Klemm P., Schembri M., Mărild S., Svanborg C. (1996). Type 1 fimbrial expression enhances *Escherichia coli* virulence for the urinary tract. Proc. Natl. Acad. Sci. USA.

[49] Nagahori N., Lee R.T., Nishimura S.-I., Pagé D., Roy R., Lee Y.C. (2002). Inhibition of adhesion of Type 1 fimbriated *Escherichia coli* to highly mannosylated ligands. ChemBioChem.

[50] Yan X., Sivignon A., Yamakawa N., Crepet A., Travelet C., Borsali R., Dumych T., Li Z., Bilyy R., Deniaud D. (2015). Glycopolymers as antiadhesives of E. coli strains inducing inflammatory bowel diseases. Biomacromolecules.

[51] Zheng L., Luo Y., Chen K., Zhang Z., Chen G. (2020). Highly branched gradient glycopolymer: Enzyme-assisted synthesis and enhanced bacteria-binding ability. Biomacromolecules.

[52] Luo Y., Gu Y., Feng R., Brash J., Eissa A.M., Haddleton D.M., Chen G., Chen H. (2019). Synthesis of glycopolymers with specificity for bacterial strains via bacteria-guided polymerization. Chem. Sci..

[53] Cramer J., Lakkaichi A., Aliu B., Jakob R.P., Klein S., Cattaneo I., Jiang X., Rabbani S., Schwardt O., Zimmer G. (2021). Sweet drugs for bad bugs: A glycomimetic strategy against the DC-SIGN-mediated dissemination of SARS-CoV-2. J. Am. Chem. Soc..

[54] Watanabe Y., Allen J.D., Wrapp D., Mclellan J.S., Crispin M.J.S. (2020). Site-specific glycan analysis of the SARS-CoV-2 spike. Science.

[55] Becer C.R., Gibson M.I., Geng J., Ilyas R., Wallis R., Mitchell D.A., Haddleton D.M. (2010). High-affinity glycopolymer binding to human DC-SIGN and disruption of DC-SIGN interactions with HIV envelope glycoprotein. J. Am. Chem. Soc..

[56] Zhang Q., Su L., Collins J., Chen G., Wallis R., Mitchell D.A., Haddleton D.M., Becer C.R. (2014). Dendritic cell lectin-targeting sentinel-like unimolecular glycoconjugates to release an anti-HIV drug. J. Am. Chem. Soc..

[57] Peck K.M., Lauring A.S. (2018). Complexities of viral mutation rates. J. Virol..

[58] Sanjuan R., Domingo-Calap P. (2016). Mechanisms of viral mutation. Cell. Mol. Life Sci..

[59] Duffy S. (2018). Why are RNA virus mutation rates so damn high?. PLoS Biol..

[60] Griffiths C., Drews S.J., Marchant D.J. (2017). Respiratory syncytial virus: Infection, detection, and new options for prevention and treatment. Clin. Microbiol. Rev..

[61] Suthar M.S., Diamond M.S., Gale M. (2013). West nile virus infection and immunity. Nat. Rev. Microbiol..

[62] Jung K., Saif L.J. (2015). Porcine epidemic diarrhea virus infection: Etiology, epidemiology, pathogenesis and immunoprophylaxis. Vet. J..

[63] Xu D., Esko J.D. (2014). Demystifying heparan sulfate–protein interactions. Annu. Rev. Biochem..

[64] Katoh M. (2016). FGFR inhibitors: Effects on cancer cells, tumor microenvironment and whole-body homeostasis (review). Int. J. Mol. Med..

[65] Zhang G.-L., Ye X.-S. (2018). Synthetic glycans and glycomimetics: A promising alternative to natural polysaccharides. Chem. Eur. J..

[66] Soria-Martinez L., Bauer S., Giesler M., Schelhaas S., Materlik J., Janus K., Pierzyna P., Becker M., Snyder N.L., Hartmann L. (2020). Prophylactic antiviral activity of sulfated glycomimetic oligomers and polymers. J. Am. Chem. Soc..

[67] Lees W.J., Spaltenstein A., Kingery-Wood J.E., Whitesides G.M. (1994). Polyacrylamides bearing pendant.alpha.-sialoside groups strongly inhibit agglutination of erythrocytes by influenza A virus: Multivalency and steric stabilization of particulate biological systems. J. Med. Chem..

[68] Liu H.-P., Meng X., Yu Q., Tao Y.-C., Xu F., He Y., Yu P., Yang Y. (2018). Synthesis of S-sialyl polymers as efficient polyvalent influenza inhibitors and capturers. J. Carbohydr. Chem..

[69] Li G., Ma W., Mo J., Cheng B., Shoda S.I., Zhou D., Ye X.S. (2021). Influenza virus precision diagnosis and continuous purification enabled by neuraminidase-resistant glycopolymer-coated microbeads. ACS Appl. Mater. Interfaces.

[70] Nagao M., Fujiwara Y., Matsubara T., Hoshino Y., Sato T., Miura Y. (2017). Design of glycopolymers carrying sialyl oligosaccharides for controlling the interaction with the influenza virus. Biomacromolecules.

[71] Matsuoka K., Kaneshima T., Adachi R., Sasaki J., Hashiguchi T., Koyama T., Matsushita T., Hatano K. (2021). Preparation of glycopolymers having sialyl alpha2 --> 3 lactose moieties as the potent inhibitors for mumps virus. Bioorgan. Med. Chem. Lett..

[72] Nagao M., Matsubara T., Hoshino Y., Sato T., Miura Y. (2019). Topological design of star glycopolymers for controlling the interaction with the influenza virus. Bioconjug. Chem..

[73] Chen W.-Y., Chang H.-Y., Lu J.-K., Huang Y.-C., Harroun S.G., Tseng Y.-T., Li Y.-J., Huang C.-C., Chang H.-T. (2015). Self-assembly of antimicrobial peptides on gold nanodots: Against multidrug-resistant bacteria and wound-healing application. Adv. Funct. Mater..

[74] Li Y., Ma X., Zhang J., Pan X., Li N., Chen G., Zhu J. (2022). Degradable Selenium-Containing Polymers for Low Cytotoxic Antibacterial Materials. ACS Macro Lett..

[75] Liu G., Xu Z., Dai X., Zeng Y., Wei Y., He X., Yan L.-T., Tao L. (2021). De Novo Design of Entropy-Driven Polymers Resistant to Bacterial Attachment via Multicomponent Reactions. J. Am. Chem. Soc..

[76] Wallis R.S., O’Garra A., Sher A., Wack A. (2022). Host-directed immunotherapy of viral and bacterial infections: Past, present and future. Nat. Rev. Immunol..

[77] McCulloch T.R., Wells T.J., Souza-Fonseca-Guimaraes F. (2022). Towards efficient immunotherapy for bacterial infection. Trends Microbiol..

[78] Gao Y., Wang W., Yang Y., Zhao Q., Yang C., Jia X., Liu Y., Zhou M., Zeng W., Huang X. (2022). Developing next-generation protein-based vaccines using high-affinity glycan ligand-decorated glyconanoparticles. Adv. Sci..

[79] Zhang Y., Wu L., Li Z., Zhang W., Luo F., Chu Y., Chen G. (2018). Glycocalyx-mimicking nanoparticles improve anti-PD-L1 cancer immunotherapy through reversion of tumor-associated macrophages. Biomacromolecules.

[80] Liu Q., Jiang S., Liu B., Yu Y., Zhao Z.A., Wang C., Liu Z., Chen G., Chen H. (2019). Take immune cells back on track: Glycopolymer-engineered tumor cells for triggering immune response. ACS Macro Lett..

